# PEG-Albumin Plasma Expansion Increases Expression of MCP-1 Evidencing Increased Circulatory Wall Shear Stress: An Experimental Study

**DOI:** 10.1371/journal.pone.0039111

**Published:** 2012-06-14

**Authors:** C. Makena Hightower, Beatriz Y. Salazar Vázquez, Seetharama A. Acharya, Shankar Subramaniam, Marcos Intaglietta

**Affiliations:** 1 Department of Bioengineering, University of California San Diego, La Jolla, California, United States of America; 2 Department of Experimental Medicine, Faculty of Medicine, Universidad Nacional Autónoma de México, Hospital General de México, México D.F., Mexico; 3 Department of Medicine, Physiology and Biophysics, Albert Einstein College of Medicine, Bronx, New York, United States of America; University of Sao Paulo – USP, Brazil

## Abstract

Treatment of blood loss with plasma expanders lowers blood viscosity, increasing cardiac output. However, increased flow velocity by conventional plasma expanders does not compensate for decreased viscosity in maintaining vessel wall shear stress (WSS), decreasing endothelial nitric oxide (NO) production. A new type of plasma expander using polyethylene glycol conjugate albumin (PEG-Alb) causes supra-perfusion when used in extreme hemodilution and is effective in treating hemorrhagic shock, although it is minimally viscogenic. An acute 40% hemodilution/exchange-transfusion protocol was used to compare 4% PEG-Alb to Ringer’s lactate, Dextran 70 kDa and 6% Hetastarch (670 kDa) in unanesthetized CD-1 mice. Serum cytokine analysis showed that PEG-Alb elevates monocyte chemotactic protein-1 (MCP-1), a member of a small inducible gene family, as well as expression of MIP-1α, and MIP-2. MCP-1 is specific to increased WSS. Given the direct link between increased WSS and production of NO, the beneficial resuscitation effects due to PEG-Alb plasma expansion appear to be due to increased WSS through increased perfusion and blood flow rather than blood viscosity.

## Introduction

Plasma expanders are used to remedy the volume component of blood loss until blood transfusion becomes necessary for the maintenance of oxygen carrying capacity. Both colloidal and crystalloid based solutions are used for this purpose. Their application dilutes blood, lowering its viscosity and increasing cardiac output, and therefore, blood flow. Within limits, this effect maintains oxygen delivery capacity as fewer red blood cells (RBCs) circulate more rapidly maintaining RBC flux. However, increased flow velocity does not compensate for the decreased blood viscosity in maintaining vessel wall shear stress (WSS). As a consequence production of nitric oxide (NO) by the vessel wall is diminished [1] causing vasoconstriction, which partially negates the increase in flow due to lower blood viscosity.

In contrast to presently available passive plasma expanders, a new approach to volume expansion is based on active plasma expanders [2]. These fluids increase plasma viscosity in hemodilution maintaining and increasing WSS in the microcirculation, promoting the release of NO, causing vasodilatation. This mechanism in combination of the lower blood viscosity due to hemodilution significantly increases cardiac output causing a state of supra-perfusion. Furthermore, this combination of effects facilitates transmission of central blood pressure to the microcirculation [3], maintaining functional capillary density, a parameter shown to improve survival during blood losses [4], [5].

Conjugation of human serum albumin (HSA) with polyethylene glycol (PEG) [6], [7] yields the colloid PEG-Albumin (PEG-Alb) that has the same supra-perfusion properties as viscogenic plasma expanders, such as alginate or dextran 500 kDa, however, Extension Arm Facilitated (EAF) PEG-Alb is significantly less viscous. PEGylation confers to albumin several desirable plasma expanding properties. It increases the molecular dimensions, i.e., hydrodynamic volume six to eight times more efficiently than a comparable mass of protein [8] and lowers the biological reactivity to colloids, increases plasma half-life, lowers immunoreactivity, and it appears to virtually eliminate thrombogeneicity. As a consequence PEG-Alb has consistently yielded better resuscitation outcomes when compared to other similar plasma expanders in experimental models of extreme hemodilution [7], hemorrhagic shock [6], [9], and endotoxemia [10].

Maintenance of high levels of perfusion found with PEG-Alb resuscitation cannot be solely attributed to its viscogenic properties contributing to increased WSS, since it is minimally viscogenic [7] once diluted in blood. Increased WSS could be due to increased flow, an effect that probably differs between organs and tissue types and not readily evidenced by conventional microcirculatory studies. Other mechanisms proposed are direct physical interactions of the PEG-shell of PEG-Alb with the endothelium, activation of the endothelium derived vasodilator response [11] and PEG-Alb’s enhanced capacity to transport NO as nitroso thiols [12]. However, experimental observations and molecular characteristics do not evidence these effects nor do they explain the superiority of PEG-Alb.

In our present study we analyze the differences in cytokine expression following *in vivo* exposure to plasma expanders to determine if PEG-Alb effects are related to the WSS/NO mechanism. The PEG-Alb used in this study is generated by a new approach to the PEGylation of proteins termed Extension Arm Facilitated (EAF) PEGylation. This process engineers a zone of extension arms, nearly 1 nm in thickness, between the outer PEG-shell and the protein core. It has been suggested that the intermediary zone of extension arms functions as a shock absorber that maximizes shielding of the protein core from macro-environmental effects, while minimizing the structural perturbations of the protein core from PEG protein interactions [5], [13]. We compare cytokine expression due to 4% EAF PEG-Alb application with the effects of plasma expanders that do not show the supra-perfusion effect using an acute hemodilution/exchange-transfusion (AHET) experimental protocol. We principally focus on monocyte chemotactic protein-1 (MCP-1), which is produced by the endothelium when WSS increases [14], [15], [16].

## Results

Preliminary investigations (n=1 per plasma expander, data not included) were completed in individual animals following a 10% top load-hypervolemic infusion prior to AHET investigations. These data were used to assess the variation and timing in cytokine production among the plasma expanders assessed prior to the AHET investigation.

During the 40% AHET experimental protocol animals were randomly assigned to 1 of 2 observation endpoints, 0.5 or 2 hrs following the infusion of individual plasma expanders: Ringer’s Lactate (RL; Baxter Healthcare Corporation Deerfield, IL), Dextran (Dex; 70 kDa molecular weight; B. Braun Medical, Inc., Irvine, CA), 6% Hetastarch (6Ht; 670 kDa average molecular weight; Hospira, Inc., Lake Forest, IL), or 4% EAF PEG-Alb (developed at Albert Einstein College of Medicine, Bronx, NY).

Hierarchical clustering analysis was applied to AHET datasets (n=2 per plasma expander/time point). The variability between each of the two AHET samples analyzed according to plasma expander and observation endpoint ranged from 0.1 to 65.9%. However, the variability between AHET samples, according to MCP-1 and the macrophage inflammatory proteins (MIP), MIP-1α and MIP-2, amongst the plasma expanders only ranged from 6.1 to 6.9%.

### Cytokine Analysis

**Figure 1:**
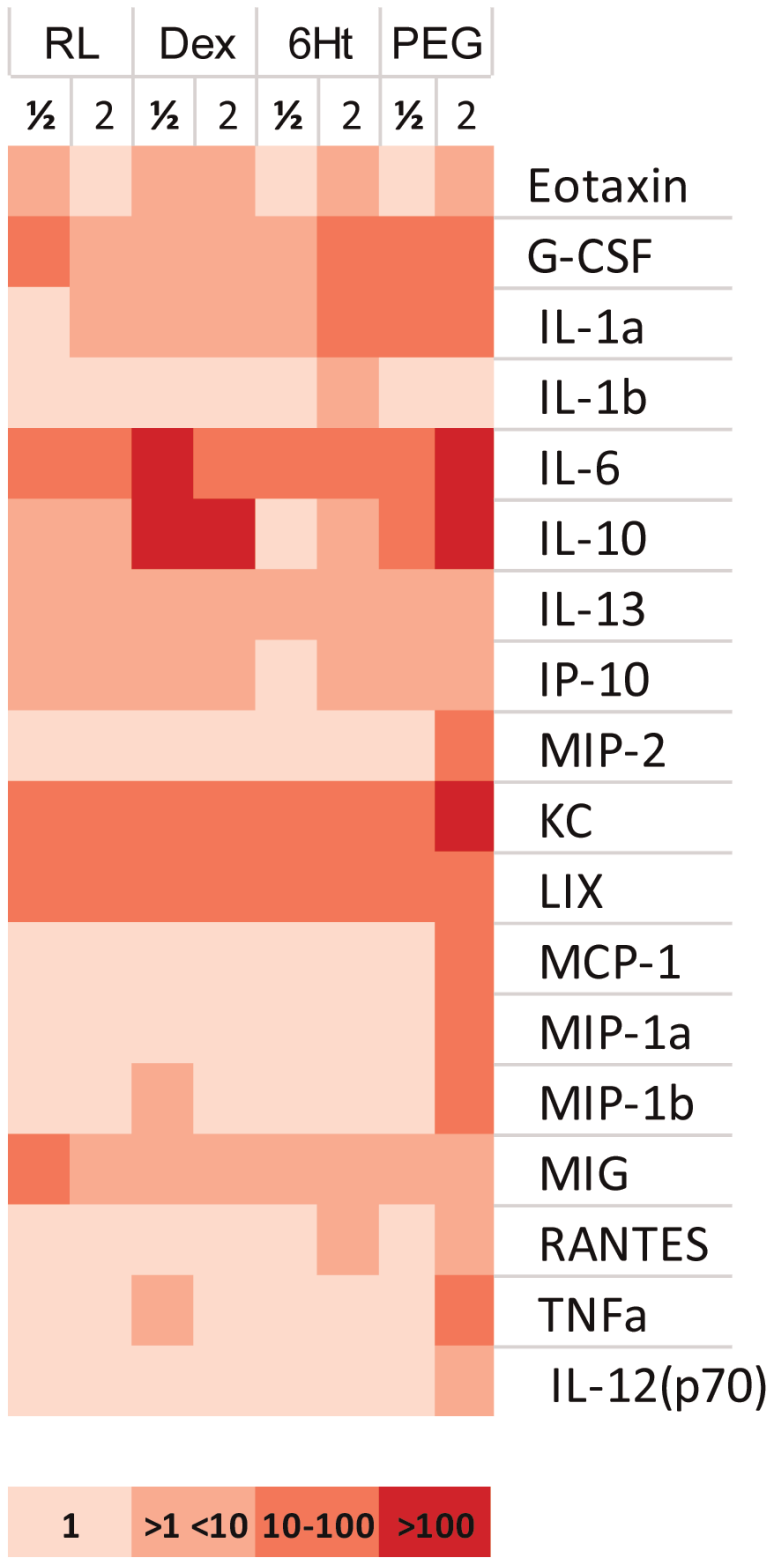
Acute hemodilution/exchange transfusion cytokines Heat Map. Expression levels of individual cytokines corresponding to the ½ and 2 hr post 40% exchange transfusion of the plasma expanders: RL, Ringer’s Lactate; Dex, Dextran 70 kDa; 6Ht, 6% Hetastarch; PEG, 4% EAF PEG-Alb. Cytokine post infusion expression levels were normalized to control levels. 1 signifies that there was no change relative to control. The remainder colors bracket multiples of control concentration. Specific values for the only cytokines to have enhanced expression due to one plasma expander 2 hr post infusion are: MCP-1, 10**×** control; MIP-1α, 14**×** control; and, MIP-2, 36**×** control.

**Figure 2:**
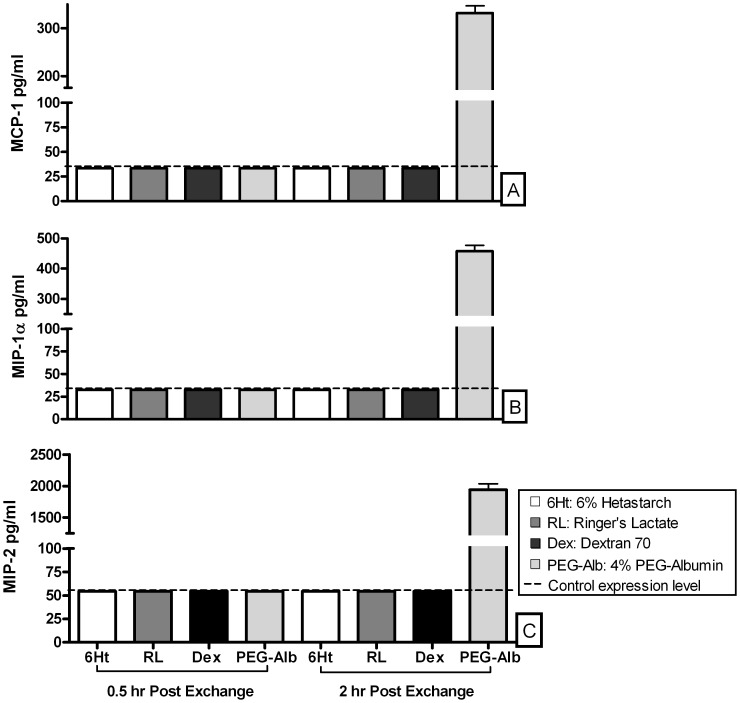
Acute hemodilution/exchange transfusion. Expression of (A) MCP-1, (B) MIP-1α, and (C) MIP-2 chemokines, 0.5 and 2 hrs post exchange transfusion of individual plasma expanders, 6% Hetastarch, Ringer’s Lactate, Dextran 70, or EAF PEG-Alb. EAF PEG-Alb was the only plasma expander to cause expression to exceed control levels at either the 0.5 or 2 hr assessment point following hemodilution.

Hemodilution with 4% EAF PEG-Alb increased the concentration of all cytokines (Eotaxin, G-CSF, IL-10, IL-12 (p70), IL-13, IL-1α, IL-6, IP-10, KC, LIX, MCP-1, MIG, MIP-1α, MIP-1β, MIP-2, RANTES, and TNF-α) 0.5 and/or 2 hr post exchange, compared to control animals (Figure 1). The level of production of MCP-1, MIP-1α, and MIP-2, was highest in animals hemodiluted with 4% EAF PEG-Alb, 2 hr following exchange transfusion (Figure 2).

### Blood Viscosities

Blood viscosity of 40% hemodiluted blood for each plasma expander group was: Hetastarch 3.1 cP, Ringer’s Lactate 2.1 cP, Dextran 70 3.8 cP and 4% EAF PEG-Alb 3.1 cP.

## Discussion

The principal result of this study is that 2 hr after 40% exchange transfusion in CD-1 mice 4% EAF PEG-Alb uniquely increased MCP-1, MIP-1α, and MIP-2 expression by comparison to Ringer’s Lactate, Dextran70, and 6% Hetastarch.

Expression of the MCP-1 gene *in vitro* is a marker for shear stress augmentation in endothelial cell culture investigations [14], [16], [17], [18] that peaks 1.5 hr following the initial rise in shear stress [14], a time course for gene up-regulation similar to that found in our present study. Therefore, our result evidences that 4% EAF PEG-Alb plasma expansion significantly increases WSS as compared to the other plasma expanders of comparable viscosity. This might explain the supra-perfusion caused by EAF-PEG-Alb since measurement of NO concentration in the microvascular vessel wall with micro-electrodes show that increased WSS due to high viscosity plasma expander hemodilution significantly increases NO concentration, leading to the vasodilator effects necessary for increasing perfusion [1].

MCP-1, MIP-1α, and MIP-2 are generally viewed as proinflammatory and are associated with vascular trauma and cardiovascular related diseases that present leukocyte activation followed by vascular transmigration [19], [20]. However, these phenomena are not present or evident in the current investigations. The short circulation half-life (<24 hr) of the plasma expanders, and the association of MCP-1 with angiogenic molecular pathways [21], [22], although a process not specifically related to plasma expansion, further supports the conclusion that the significant up-regulation of MCP-1 by 4% EAF PEG-Alb is caused by increased WSS in the circulation, an effect absent when using conventional plasma expanders. Notably, RANTES, a cytokine also associated with the increase in WSS [23] was only expressed by the low viscosity plasma expanders 6% Hetastarch (1.6**×** control) and 4% EAF PEG-Alb (1.8**×** control).

Additionally, the advantageous effects of MIP-1α and MIP-2 concern their stimulatory influence on hematopoietic progenitor (HPC) and stem cell (HSC) mobilization from the bone marrow to peripheral blood, [24], [25], [26] to be used for autologous or allogeneic transplantation. However, recent investigations have built on the assumption that HSCs have the capacity, as with other tissue derived stems cells, to contribute to the regeneration of non-native tissue [27]. Findings reported by Grant M. et al. indicated that HSCs and HPCs can in fact be a major source of endothelial cells used for ischemia induced neovascularization [28]. Previous experimental protocol time scales [6], [7], [10] do not allow for the significant benefits reported for 4% EAF PEG-Alb to be related to neovascularization. However, HSCs and HPCs plasticity allowing their incorporation into the surrounding tissue, as well as the endothelium is believed to initiate tissue preservation and/or regeneration signaling, a driving factor in the conservation and/or reestablishment of tissue perfusion following injury. Reportedly, stimulation of HSC and HPC mobilization following MIP-1α and MIP-2 is rapid and dose dependent [26], [29]. Thus, 4% EAF PEG-Alb stimulated mobilization of HSC and HPC is possible within previous experimental protocol time frames [6], [7], [10].

EAF PEG-Alb also increases the concentration the hematopoietic cytokines G-CSF (49× control) and IL-6 (353× control) 2 hr after exchange transfusion. These cytokines function as hormones that regulate blood cell production and are used in the management of anemia and neutropenia. This suggests that EAF PEG-Alb signals hematopoiesis, an effect also evidenced to a lesser extent by exchange transfusion with 6% Hetastarch, driven by the reduction in oxygen carrying capacity due to hemodilution.

The increase in cytokine expression following exchange transfusion with each plasma expander may most likely be also in part due to a general response of the organism to the alteration of blood composition and the introduction of a foreign material into the circulation. The increased expression of proinflammatory and inflammatory cytokines IL1a, IL1b, IL-6, IL-10, IL12, IL13, TNFα and Eotaxin by EAF PEG-Alb relative to the other plasma expanders is probably also related to RL, Dextran 70 and Hetarstarch being produced in GMP conditions for human use, while EAF PEG-Alb was produced in clean laboratory conditions. Cytokines should return to baseline levels within a time frame commensurate with the half-life of plasma expanders, a question to be addressed in further studies.

In conclusion, plasma expansion with 4% EAF PEG-Alb is associated with the elevation of MCP-1, a chemokine specifically related to increased shear stress on the endothelium, [14], [16], [17], [18] which increases NO production causing vasodilatation. Lowered blood viscosity due to hemodilution in combination with the increase in NO that should be the consequence of increased WSS promotes vasodilatation and a condition of enhanced perfusion, which maintains WSS and endows EAF PEG-Alb with highly beneficial plasma expansion properties. Moreover, 4% EAF PEG-Alb significantly increases expression of hemopoietic cytokines potentially signaling the bone marrow to supplement the deficiency of blood cells.

## Materials and Methods

### Ethics Statement

The Guide for the Care and Use of Laboratory Animals (US National Research Council, 1996) was followed for animal handling and provided care. The protocol was approved by the University of California, San Diego Animal Subjects Committee (Permit Number: S09221). All surgery was performed under sodium pentobarbital anesthesia, and every effort was made to minimize suffering.

### Polyethylene Glycol Surface Decorated Human Serum Albumin (PEG-albumin)

EAF PEGylation of albumin was carried out as described previously [5]. Briefly, lyophilized preparations of albumin from Sigma Aldrich were subjected to EAF PEGylation at a temperature of 4°C and a protein concentration of 0.5 mM, using 10 mM maleimidophenyl PEG 5K (custom synthesized), in the presence of 5 mM 2-IT. The hexaPEGylated albumin thus generated had molecular weight 95–100 kDa and was then purified through tangential flow filtration and concentrated to a 4 g% solution and stored at −80°C.

### Animal Model and Preparation

Investigations were performed in CD-1 mice (Charles River), 6 to 7 weeks of age, weighing 18–20 grams, using a simplified version of the window chamber model used for microvascular studies in unanesthetized animals. This model was used to support the catheters and prevent the mice from damaging the catheters, while maintaining them in place. Chamber and vascular catheterization surgeries were performed under general anesthesia, 50 mg/kg i.p. injections, as described previously [30], [31] with the modification that the surgical exposure of the tissue and its microcirculation was not implemented. Following chamber implantation, animals were allowed a minimum 2 day recovery period prior to catheterization. Animals were anesthetized for carotid artery and jugular vein catheter implantation (polyethylene-50), following chamber assessment ruling out the presence of edema, bleeding, or signs of infection.

### Inclusion Criteria

All surgical sites of investigated animals were free from signs of edema or bleeding on the day of experimentation. Animals needed also to fulfill baseline systemic parameter requirements: mean arterial pressure (MAP) above 80 mmHg, heart rate (HR) above 320 beats/min, systemic hematocrit (Hct) above 40%, and arterial oxygen tension above 50 mmHg.

### Systemic Parameters

MAP and HR were monitored using a Biopac acquisition system (MP 150; Biopac Systems, Inc., Santa Barbara, CA). Systemic Hct was measured from arterial blood collected in heparinized microcapillary tubes and centrifuged.

### Experimental Groups

Preliminary investigations of plasma expanders were completed in individual animals following a 10% top load-hypervolemic infusion protocol (THI), prior to AHET investigations.

During the 40% AHET experimental protocol animals were randomly assigned to 1 of 2 observation endpoints, 0.5 or 2 hrs following the infusion of individual plasma expanders: Ringer’s Lactate (RL; Baxter Healthcare Corporation Deerfield, IL), Dextran (Dex; 70 kDa molecular weight; B Braun Medical, Inc. Irvine, CA), 6% Hetastarch (6Ht; 670 kDa average molecular weight; Hospira, Inc. Lake Forest, IL), or 4% EAF PEG-Alb (developed at Albert Einstein College of Medicine, Bronx, NY).

### Experimental Setup and Acute Hemodilution/Exchange-transfusion

Unanesthetized animals were placed in a restraining tube and given a 30–60 min adjustment period. Baseline measurements were then taken (MAP, HR, and Hct). The volume of the 40% AHET was calculated as a percentage of the animal’s total blood volume, estimated as 6% of body weight. Animals were observed through their assigned end time points. Upon the conclusion of observation periods 30 to 50% of the animal’s total blood volume was collected for cytokine assay. Blood of control animals was collected following baseline measurements, without having been exposed to the infusion/transfusion of fluids and the AHET protocol.

### Cytokine Analysis

Whole blood was collected, without anticoagulant from the arterial catheter. Blood was allowed to clot for 30 min and was then centrifuged for 10 min at 1000 g. Serum supernatants were removed immediately, placed in Eppendorf tubes, and stored at −80°C until analyzed. Samples were assayed for 31 analytes (Eotaxin, G-CSF, GM-CSF, IFN-γ, IL-10, IL-12 (p40), IL-12 (p70), IL-13, IL-15, IL-17, IL-1α, IL-1β, IL-2, IL-3, IL-4, IL-5, IL-6, IL-7, IP-10, KC, LIF, LIX, M-CSF, MCP-1, MIG, MIP-1α, MIP-1β, MIP-2, RANTES, TNF-α, and VEGF) using the MILLIPLEX™ MAP Mouse Cytokine/Chemokine Panel (BioMarker Services, Millipore, St. Charles, MO).

### Blood Viscosity Measurements

Hemodiluted blood viscosity was determined *in vitro*. Whole blood collected from donor mice was mixed to the appropriate 60∶40 ratio with each of the individual plasma expanders. Viscosity measurements were performed with a cone/plate viscometer (model DV-II; Brookfield; Middelboro, MA), CPE-40 cone spindle at 37°C, and a shear rate of 200 s^−1^, representative of the prevalent shear rates found during normal flow states of the microvasculature [32], [33].

### Data Analysis

Data analysis was performed using the dChip 2008 (build date 8th May 2008) platform and/or GraphPad Prism version 4 software (La Jolla, CA).
